# The Role of β-Arrestin Proteins in Organization of Signaling and Regulation of the AT1 Angiotensin Receptor

**DOI:** 10.3389/fendo.2019.00519

**Published:** 2019-08-06

**Authors:** Gábor Turu, András Balla, László Hunyady

**Affiliations:** ^1^Department of Physiology, Faculty of Medicine, Semmelweis University, Budapest, Hungary; ^2^MTA-SE Laboratory of Molecular Physiology, Semmelweis University, Hungarian Academy of Sciences, Budapest, Hungary

**Keywords:** AT1 receptor, angiotensin II, signaling, biased agonism, arrestin

## Abstract

AT1 angiotensin receptor plays important physiological and pathophysiological roles in the cardiovascular system. Renin-angiotensin system represents a target system for drugs acting at different levels. The main effects of ATR1 stimulation involve activation of Gq proteins and subsequent IP3, DAG, and calcium signaling. It has become evident in recent years that besides the well-known G protein pathways, AT1R also activates a parallel signaling pathway through β-arrestins. β-arrestins were originally described as proteins that desensitize G protein-coupled receptors, but they can also mediate receptor internalization and G protein-independent signaling. AT1R is one of the most studied receptors, which was used to unravel the newly recognized β-arrestin-mediated pathways. β-arrestin-mediated signaling has become one of the most studied topics in recent years in molecular pharmacology and the modulation of these pathways of the AT1R might offer new therapeutic opportunities in the near future. In this paper, we review the recent advances in the field of β-arrestin signaling of the AT1R, emphasizing its role in cardiovascular regulation and heart failure.

## Introduction

AT1 angiotensin II receptor (AT1R) belongs to the G protein-coupled receptor (GPCR) family of membrane receptors and is activated by the octapeptide hormone, angiotensin II (AngII). AngII is the main effector of the renin-angiotensin system (RAS), which has pleiotropic effects in the cardiovascular system and salt-water balance regulation. As such, the RAS is an important target in the treatment of various cardiovascular diseases. AT1R blockers (ARBs) and angiotensin-converting enzyme inhibitors are widely used for the treatment of hypertension. Moreover, ARBs have beneficial effects beyond lowering the blood pressure, by preventing cardiovascular organ injuries (1). AngII can bind and activate two types of angiotensin receptors (type 1 and type 2 angiotensin receptors). Some cells contain AT2-receptors that mostly act via Gi and tyrosine phosphatases showing somewhat opposite effects to the AT1R-mediated cellular responses (2). However, the main physiological and pathological effects of RAS are mediated by AT1R. Initially, G protein activation was considered to be the only signal transduction process, which mediates the effects of AngII via AT1R. After AngII binding to AT1R, a heterotrimeric G protein, Gq mediates the hydrolysis of PtdIns(4,5)P2 by phosphoinositide-specific phospholipase Cβ resulting in a generation of second messengers. In addition, AT1R can also couple to Gi/o and G12/13 proteins leading to inhibition of adenylyl cyclase, activation of phospholipase D, Rho-kinase, and regulation of Ca^2+^ channels (3, 4).

Following their activation, GPCRs usually undergo G protein kinase (GRK) dependent phosphorylation on serine and threonine residues on their C-terminal tails. This phosphorylation leads to binding of β-arrestin proteins, which shut down G protein activation and target the receptors toward internalization. During the last two decades it became increasingly evident that, in addition to G proteins, β-arrestins can also mediate signaling events. β-arrestin proteins serve as scaffold proteins, bringing together the players of different protein kinase cascades, and instead of turning it off, they switch the signaling toward different routes (5). Like many other GPCRs, AT1R also binds β-arrestins, and β-arrestins regulate their internalization (6–8) Since the recognition of the role of β-arrestins in AT1R internalization, AT1R served as one of the most studied model for β-arrestin-dependent signaling, which has led to a completely new field in molecular pharmacology with the potential of development of new drugs, which may exploit the possibilities in selective activation of GPCR-activated signaling pathways.

## Structural Requirements for Internalization and β-arrestin Binding to the AT1R

The binding of agonists to G protein-coupled receptors initiate the “classical,” G protein-mediated pathway, which results in the production of second messenger molecules. Termination of signaling has a key role in the regulation of the kinetics of receptor function. Mechanisms regulating receptor function include several consecutive or parallel processes, such as desensitization, internalization into intracellular vesicles, and degradation of the receptors. Receptor internalization is regulated by phosphorylation by GPCR kinases, which promotes the β-arrestin binding leading to desensitization, internalization and altered signaling of GPCRs (9). The role of a serine-threonine rich domain in the C-terminus of AT1R for angiotensin receptor internalization was first described in the laboratory of Kevin J. Catt (10–12). It was initially discovered with mutational and truncation analysis of AT1R that Thr332-Lys333-Met334-Ser335-Thr336-Leu337-Ser338 amino acids, particularly the Ser335-Thr336-Leu337 motif on the C-terminus of the receptor are required for receptor internalization (10). A large number of serine and threonine amino acids in this region suggested the possible involvement of phosphorylation in its regulation. Indeed, Thr332, Ser335, Thr336, and Ser338 amino acids have been later identified as phosphorylation sites that regulate endocytosis (12–14), and involvement of β-arrestins in receptor internalization was verified (6, 15). The same serine and threonine amino acids were identified as the region critical for stable interaction between AT1R and β-arrestins following AngII stimulation (15, 16). On β-arrestin, two critical lysins (K11 and K12 in β-arrestin2), the phosphate-binding residues, are responsible for the stable interaction (17–22). It turns out, that the interaction between the serine/threonine-rich region and these two lysins (the “stability lock”) is responsible for the conformational rearrangement in βarrestin2 protein, leading to recruitment of the members (23, 24) and activation of Erk MAPK cascade (16). Interestingly, not only receptor activation, but also PKC mediated phosphorylation of the inactive, unliganded AT1R alone leads to recruitment of β-arrestin2, receptor internalization and scaffolding of the signaling complex (24).

## The Concept of Biased Agonism

Ligand binding to a plasma membrane receptor can initiate several parallel signal transduction pathways leading to various responses in the cell. Ligands of the plasma membrane receptors were originally classified as agonists and antagonists (or more recently inverse agonists). However, several ligands are capable of selectively initiating one or more of distinct signal transduction pathways coupled to one receptor, which phenomenon has been referred to as “biased agonism” or “functional selectivity.” It has been revealed that the biased agonism is an important feature of several members of the GPCR superfamily, and it is proposed that the biased agonists can serve as new therapeutic agents (25). Some of the GPCR ligands can selectively couple the given receptor to the different downstream signaling events, including G protein activation and β-arrestin-mediated signaling, leading to altered signaling patterns compared to the natural agonist. The background of this phenomenon is that the variant ligands cause distinct conformational changes in the structure of the receptor. The different conformations can lead to dissimilar interaction capabilities with G proteins and to other partners. Another layer of complexity is introduced by the recognition of different phosphorylation patterns on the receptor C-terminus. It has been recognized, that different receptors bind arrestins with different affinities, some of which form stable (called class B receptors) whereas others form loose interactions with β-arrestins (called class A receptors) (26). Interaction stability and arrestin activation seem to be determined by specific patterns of phosphorylated serine/threonine amino acids (22, 27). Moreover, different kinases may phosphorylate different residues on the C-terminus, resulting in slightly different outcomes regarding internalization, signaling and activated β-arrestin conformations. This phenomenon has led to the barcode theory. Slightly different active receptor conformations, different cellular expression contexts, or even simultaneous stimulation of other receptors can lead to altered phosphorylation patterns, leading to different conformations of β-arrestins, and altered signaling (28–34). AT1R phosphorylation by GRK2/3 regulates receptor endocytosis, while phosphorylation by GRK5/6 activates Erk1/2 (35). Even PKC phosphorylation can initiate β-arrestin binding and recruitment of the MAPK machinery to the AT1R, although the binding affinity is lower and the active conformation of the β-arrestin is altered (24). These data together suggest, that signaling bias is not restricted to the G-protein-β-arrestin axis, but even β-arrestin dependent signaling may be finely tuned toward distinct outcomes depending on the actual stimulation and cellular context.

Early studies had identified mutant receptors of the highly conserved Asp125Arg126Tyr127 sequence of AT1R (i.e., DRY/AAY mutation), which are not able to couple to G proteins, but still capable to internalize and bind β-arrestins (7, 36). Using this mutant AT1R, it has been demonstrated that AT1R can cause ERK 1/2 phosphorylation or Src activation in the absence of G protein activation (7, 37, 38). Furthermore, a biased AT1R agonist, [Sar1,Ile4,Ile8]-AngII (SII-AngII) was developed, which is a mutated octapeptide angiotensin analog unable to activate Gq-proteins, but still recruits β-arrestins to the AT1R and is able to evoke the internalization of the receptor (39). Thus, it acts as a biased agonist, which selectively activates β-arrestins. This octapeptide further widened the possibilities to investigate G protein-independent mechanisms. It has been shown, that this agonist can also stimulate G protein-independent mechanisms (7). It was proposed that G protein-mediated MAP kinase activation regulates nuclear targets, while the β-arrestin-mediated MAP kinases activation affects the phosphorylation of cytoplasmic proteins (40, 41). Also, compared to G-protein-dependent Erk1/2 activation, β-arrestin-dependent Erk1/2 signaling is slower, reaching its maximum after 10 min and remains active for a prolonged time (40). Later, several new AngII peptide analogs have been developed and characterized with a bias toward G protein or β-arrestin signaling (42–44). The discovery of the biased signaling has led to the recognition that AT1R may develop multiple active conformations, and this has been demonstrated by molecular dynamics simulations (45, 46) and experimentally (47–49). Conformational changes within AT1R may be followed with BRET sensors utilizing a small molecule acceptor (FlAsH molecule). When placed on different places within AT1R, different conformational changes following stimulation with distinct ligands can capture multiple activation states of the receptor (47). Different conformational states after binding of the different ligands have been further demonstrated using single molecular spectroscopy (48) and more recently using double electron-electron resonance spectroscopy in AT1R (49). The developed biased agonists serve now as widely used tools to study the biased agonism of the AT1R, and numerous of their effects on cell responses were published in recent years (50).

## Signaling Pathways Regulated by AT1R Through β-arrestin

β-arrestin-mediated mechanisms can be considered as the second wave of signal transduction events resulting in alternative outcomes. Moreover, the agonist binding of AT1R can lead to direct association with wide spectra of cytoplasmic signaling proteins, such as AT1R-associated protein (ATRAP), SHP-2, JAK2, β-arrestins, and phospholipase Cγ, which explains a plethora of AT1R generated signaling mechanisms, including JAK/STAT pathway and MAPK cascade activation (51–54). The attached proteins can initiate diverse cellular responses from the receptor-arrestin complex and can form multiprotein units, “signalosomes” (55).

One such selectively activated pathway of particular interest is the β-arrestin-dependent signaling. β-arrestin-mediated signaling events include activation of Src tyrosine kinases extracellular signal-regulated kinase 1/2 (ERK 1/2), c-Jun N-terminal kinase (JNK), mitogen-activated protein kinase (MAPK) cascades, Akt and p38 mitogen-activated protein kinases (56–59).

Besides the observation of Erk1/2 activation through β-arrestins, many other cellular functions have been discovered to be regulated through GPCRs in a non-canonical G protein-independent way. These pathways include JNK3, p38, and Akt protein kinase regulation (56), some of which have been also implicated in AT1R signaling (60, 61). The pathways may include very broad signaling routes, including the increased protein synthesis via β-arrestin-mediated Mnk1, eIF4E, and ERK1/2 activation (62). The arrestin-biased ligand, SII-AngII is also able to increase the aldosterone synthesis in adrenocortical zona glomerulosa cells via β-arrestin, in addition to the AngII-induced Gq protein-dependent aldosterone production, which can lead to adverse cardiac remodeling and heart failure progression (63). Several proteomic and genetic studies were carried out to investigate the short and long term effects of the treatment of SII-AngII using both AT1R overexpressing cells and primary cell cultures. It has been demonstrated that the SII-AngII could initiate a robust G protein-independent signaling network, and signaling pathways significantly differ than the AngII induced cellular responses. Kendall et al. demonstrated that the SII-AngII treatment changed the phosphorylation state of numerous downstream proteins, such as protein phosphatase 2A and prostaglandin E synthase 3 (64). The inhibitory phosphorylation of protein phosphatase 2A results in Akt activation and phosphorylation of glycogen synthase kinase 3β, whereas the prostaglandin E synthase 3 activation increases prostaglandin production both in AT1R expressing HEK293 cells and in rat aortic vascular smooth muscle cells (64). It is very astonishing that the SII-AngII-induced phosphorylation patterns have limited overlap with those of AngII-induced phosphorylation (64), suggesting that the β-arrestin activation initiates not only spatially, but also qualitatively different signaling events. Analyzing the β-arrestin-mediated phosphoproteome after SII stimulation of AT1R revealed a plethora of changes in protein phosphorylation, including a huge number of kinases and some phosphatases as well (65). Besides the activation of different kinase cascade signaling pathways, β-arrestin-dependent regulations of ion channels have been also reported following AT1R activation, including TRPV4 (66), TRPC3 (67), and CaV1.2 (68) channels.

## Role of the β-arrestin Dependent AT1R Signaling in Cardiac Function and Heart Failure (HF)

Since the AngII is a vasopressor hormone, its production and effects are very important in the development of numerous cardiovascular diseases such as hypertension, atherosclerosis, and cardiac hypertrophy. Indeed, AT1R blockers and inhibitors of AngII production (ACE inhibitors) are now extensively used in the treatment of hypertension and other cardiovascular diseases (69, 70). Special fields of interest, where RAS plays significant roles are cardiac hypertrophy and heart failure, where AngII seems to contribute to the pathophysiology of these conditions.

Both AT1R-activation of G protein-dependent and G protein-independent mechanisms contribute to the development of cardiac hypertrophy. It was demonstrated that heart-specific overexpression of different AT1R mutants can lead to hypertrophy, moreover, the mutant AT1R, which mediates only G protein-independent signaling mechanisms, caused greater cardiac hypertrophy but less apoptosis and fibrosis than overexpression of wild-type AT1R (71). Contrary, in vascular smooth cells (VSMCs) only the G protein-dependent mechanism of AT1R, mediated by EGF receptor transactivation and Rho kinase activation, seems to be important in AngII-induced hypertrophy (72). However, β-arrestin 1 dependent activation of RhoA has been demonstrated as well, and interaction of AT1R with SHP2 through Y319 residue might be involved in EGF receptor transactivation (73, 74). Interestingly, with wild type AT1Rs, cardiac hypertrophy was reported to be dependent on G protein and metalloprotease activation but did not occur after β-arrestin biased SII-AngII stimulation of AT1Rs (75). According to Aplin et al., the G protein-independent signaling supports the non-hypertrophic proliferation of rat cardiomyocytes (76, 77).

In the development of pathological cardiac hypertrophy, mechanical stress is thought to be one of the most important factors. Parallel with the discoveries of the β-arrestin-dependent signaling through AT1R, AT1R receptor was also found to be involved in stretch-induced signaling pathways in various cell types. First, it has been discovered, that in cardiomyocytes, stretch-induced Erk1/2 activation was dependent on AT1Rs (78–80). Since then, many further papers have been published with observations of mechanical stretch-induced, AT1R-mediated signaling in different cell types, including cardiomyocytes (81–85), vascular smooth muscle cells (86–89), renal podocytes (90), endothelial (91), and epidermal cells (92).

Interestingly, mechanical activation of AT1R caused increased affinity toward β-arrestin biased ligand TRV 120023 (93), suggesting stabilization of a biased active receptor conformation. Moreover, protein kinase C and GRK2, the kinase responsible for phosphorylation and subsequent β-arrestin binding of many GPCRs, has been reported to be activated upon stretch in neonatal rat ventricular myocytes (94). Indeed, hypo-osmotic stretch and mechanical stress induced β-arrestin translocation to the AT1R (88, 95). It turns out that EKR1/2 activation by mechanical stress through AT1R is β-arrestin dependent (95). Stretch-induced β-arrestin-dependent signaling may involve Erk1/2, Akt kinases, EGFR transactivation (92, 95), and Src may also play an important role in this process (96). In line with the very recent developments (see below), mechanically activated β-arrestin signaling may also involve the activation of Gi/o proteins through AT1R (97).

The involvement of AT1R- β-arrestin interplay in cardiac function has become more evident with the discovery of the beneficial effects of biased AT1R ligands on the heart contractility. RAS is typically activated in patients with HF, with increased circulating AngII levels. Anti-RAS drugs, like AT1R blockers and ACE inhibitors, have been long proven beneficial in the treatment of HF. It seems that the pathological actions of AT1R on vasoconstriction and heart remodeling are mediated through Gq-protein activation. On the contrary, studies have suggested that TRV120023 and TRV120027, a β-arrestin biased ligand of AT1R, decrease blood pressure similarly to AT1R blockers, but unlike those drugs, it improves cardiac performance, preserves cardiac stroke volume, decreases systemic vascular resistance, improves cardiac output while preserving renal functions in animal models (44, 98).

Moreover, TRV120027, in combination with furosemide, decreased preload and afterload in dog models of HF, while furosemide-induced natriuresis and diuresis were preserved (98). Infusion of biased AT1R ligand TRV120023 into mice with familial dilated cardiomyopathy increased myosin light-chain phosphorylation and improved cardiac contractility (99). Earlier studies have already shown, that SII-AngII treatment can lead to increased cardiomyocyte inotropy and lusitropy (100), and in line with that, Frank-Starling mechanism of the heart, which describes the volume load-contractility relationship of the heart, has been found to be dependent both on AT1R and β-arrestins (101). Besides Ca^2+^ sensitization, in immature mouse cardiomyocytes, TRV120027 activates CaV 1.2 Ca^2+^ channels as well through Src-family tyrosine kinases and casein kinase 2 in a β-arrestin-dependent manner (102).

After the initial promising results with biased AT1R ligands on the heart function, the first human study was reported in 2013 (103) and in the same year, the phase IIb study was initiated in patients with acute HF, and the results were reported in 2017 (104). Unfortunately, the results of this study did not confirm the beneficial effects of the biased AT1R activation, in fact, in none of the primary endpoints was observed an improved outcome. However, in this study, short time therapy was assessed; the patients were treated for only 48–96 h after within 24 h of initial presentation, and then the outcomes of acute heart failure were investigated. TRV120027 itself is an octapeptide, which has a half-life of only about a few minutes, so it has to be administered intravenously, and this restricts its long-time usability in patients. Therefore, although this study failed to justify the use of biased AT1R ligands in HF failure therapy, long-term treatment may be still beneficial. Indeed, a recent study on dilated cardiomyopathy mouse model showed improved cardiac structure and function after 3 months of TRV120067, another biased AT1R ligand treatment (105). However, TRV120067 is also a peptide, which limits its application in human patients in long-term therapy plans. The use of small molecules having β-arrestin biased activity on AT1R might be beneficial, however, to date, only one such molecule has been reported. Although troglitazone is a small molecule, potentially suitable for long-term treatments, this molecule has a very weak affinity toward AT1R, making it unattractive in *in vivo* experiments (106).

Although these experiments have very promising results, some important aspects of the RAS are often overlooked which may influence the *in vivo* results. It is widely accepted now, that AT1R can form heterodimers with other GPCRs, and the function of the heterodimers might be influenced by the active state of the AT1R. A classic example of interaction is bradykinin B2 receptor, but there are also other examples of interactions (38, 107–117). In HEK293 cells, SII-AngII inhibits B2 receptor-mediated Gq/11-dependent intracellular calcium influx when AT1R is coexpressed, showing that AT1R-biased ligands effects might be mediated not only by β-arrestins, but also by more complex interactions between different GPCRs (118). Another often overlooked aspect of the biased ligand actions is the presence of the other angiotensin II receptor *in vivo*, the AT2 angiotensin receptor (AT2R). Although the AT2R does not bind β-arrestins upon Ang II stimulation (119), it still may play role in *in vivo* effects of the AT1R biased agonists. SII-AngII, TRV120023 and TRV120027 seem to bind to this receptor as well, but their effects were mediated through AT1R in experiments where AT2 receptors were checked (44, 120, 121). However, to our knowledge, the effects of these ligands have not been thoroughly tested on AT2R.

In any case, with the better understanding of the AT1R conformation upon biased activation (45–49), and the readily availability of bioinformatical tools in drug screening (122), the discovery of high affinity drugs with small molecular weight might not be that far.

## Is β-arrestin Signaling G Protein-Independent?

**Figure 1:**
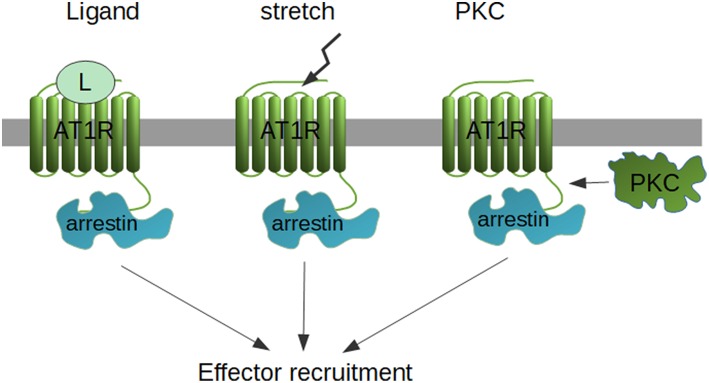
Possibilities of β-arrestin recruitment. β-arrestins are recruited to the AT1R either by activation by ligand or stretch, or by PKC-dependent phosphorylation of the unliganded receptor.

Originally, β-arrestin-mediated signaling was thought to be independent of G protein activation. In fact, AT1R-activated pathways have been usually divided into G protein-dependent and G protein-independent (e.g., β-arrestin-dependent), since AT1R mutants used in the studies and biased AT1R ligands failed to activate the usual Gq-protein signaling with IP3 and DAG production and consequent Ca^2+^ signal generation. It has challenged the concept of the clean β-arrestin dependent Erk1/2 activation when SII-AngII, the most widely used β-arrestin biased ligand, has been shown to activate Gq and Gi proteins, however with much weaker efficacy then AngII (123). In addition, this Erk1/2 activation was dependent on G protein activation. Similarly, the mechanic stretch-induced activation of Erk1/2 signaling seems to be also Gi-dependent, although in this setup, β-arrestin biased ligands (TRV12023 and TRV12026) did not require Gi coupling (97). However, very recently, using pathway-wide BRET signaling sensors, 14 different AT1R ligands have been tested, including the β-arrestin biased ones, such as TRV120027. Strikingly, all of the tested ligands activated Gi and G12 proteins with some efficacy (124), raising the possibility that some β-arrestin-dependent signaling pathways may utilize one or more G protein-dependent signaling mechanisms. Indeed, another recent study has used CRISPR-Cas9 system to switch off all G proteins, including G12/13 and used PTX to inhibit Gi (“zero functional G” setup) (125). They found, that although β-arrestin still couples to a set of GPCRs, with no functional G proteins, the Erk1/2 could not be activated. On the other hand, CRISPR deletion of β-arrestin1/2 results in various effects on Erk1/2 signaling, depending on the cell line and receptor type, it may be enhanced, inhibited or unchanged (126). These data show that the picture of β-arrestin-mediated signaling is more complex than we originally thought. The cellular background, adaptive mechanisms, β-arrestin's dual role (desensitization and activation of β-arrestin-dependent Erk1/2 signaling) lead to a very complex regulation mechanism where the end-effect on the signaling cascade will be determined on the interplay of these aspects (126). The available data suggest that during β-arrestin-signaling a complex interplay occurs between β-arrestin-signaling and kinase cascades. This model could explain the recent developments of the field. Although β-arrestin sequestrates together with all the components of the cRaf/MEK/Erk1/2 cascade, the activation of the most proximal kinase in the cascade may be kicked on by β-arrestin-independent mechanisms, which might be either a G proteins, or in more physiological settings, one of the classical, growth factor receptors (56, 127). So far, at least three different mechanisms may lead to β-arrestin coupling to the AT1R, the classical ligand-dependent way, mechanical stretch and PKC activation (Figure 1). When no other stimulus is present, β-arrestins might not be able to activate the most proximal kinases, but when they are already activated, it brings together all the pieces of the signaling cascade and orchestrates their spatiotemporal regulation.

## Concluding Remarks and Future Perspectives

We have come a long way in the recent almost 20 years since the interaction of AT1R and β-arrestin has been discovered. The interaction of these two proteins itself had a huge effect in understanding how the GPCRs function, and how are the signaling pathways regulated beside the classical G protein activation. We are at the doorstep of therapeutic exploitation of this system, and although the first attempt to cross it was not successful, recent results suggest that this was probably not our last chance. Recent advances in the field also show that there might be still a lot learn about β-arrestin-dependent signaling, and AT1R might further serve as a good model for such experiments.

## Author Contributions

All authors listed have made a substantial, direct and intellectual contribution to the work, and approved it for publication.

### Conflict of Interest Statement

The authors declare that the research was conducted in the absence of any commercial or financial relationships that could be construed as a potential conflict of interest.
